# Supplementary material for: Thermal crumpling of perforated two-dimensional sheets
Source: Nat Commun. 2017 Nov 9;8:1381. doi: 10.1038/s41467-017-01551-y (PMC5680302; doi:10.1038/s41467-017-01551-y)
Supplement: Supplementary file 1 — Supplementary Information [file 41467_2017_1551_MOESM1_ESM.pdf]

SUPPLEMENTARY FIGURE 1

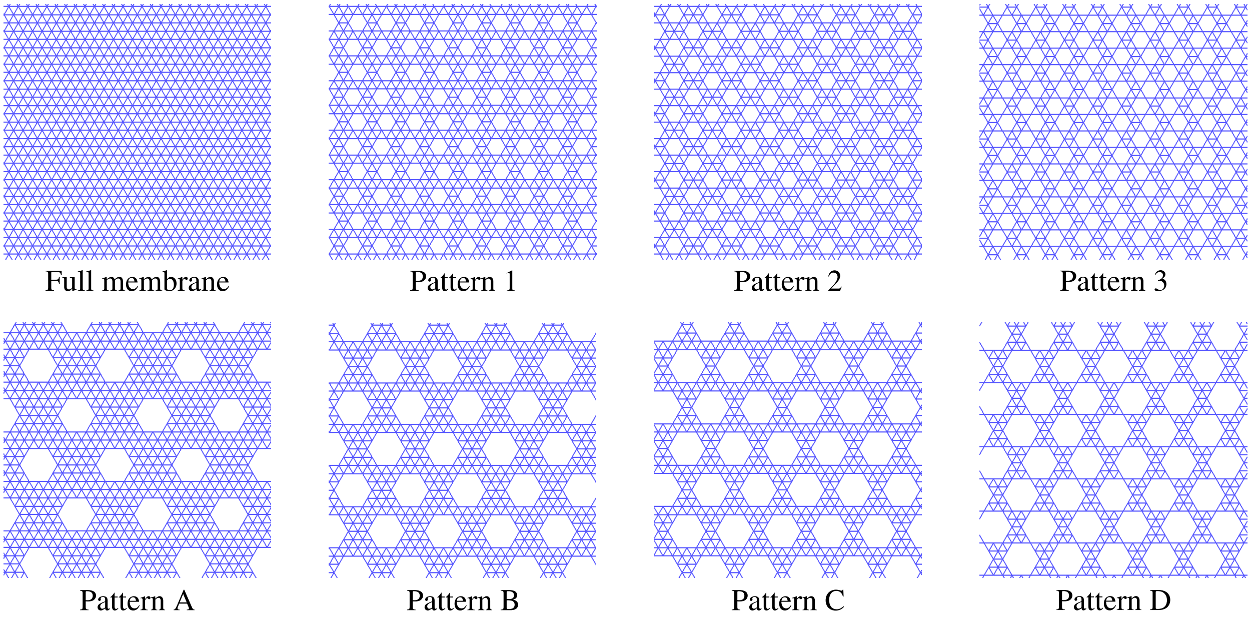

Supplementary Figure 1. List of the membrane geometries considered in this paper, using the same labels as in Fig. 3 in the main text. The top row shows the different perforation patterns with hole radius $R = 1$ (plus the full membrane). The bottom row shows the geometries wih $R = 2$. In order to see the details of these crystalline membranes, we only show a $30a \times 30a$ portion of the full lattices (of size $L \times L = 100a \times 100a$ throughout most of the paper). We use our most perforated membrane (Pattern D) for our finite-size scaling study.

SUPPLEMENTARY NOTE 1: $T = 0$ EFFECTIVE ELASTIC CONSTANTS OF THE PERFORATED MEMBRANES

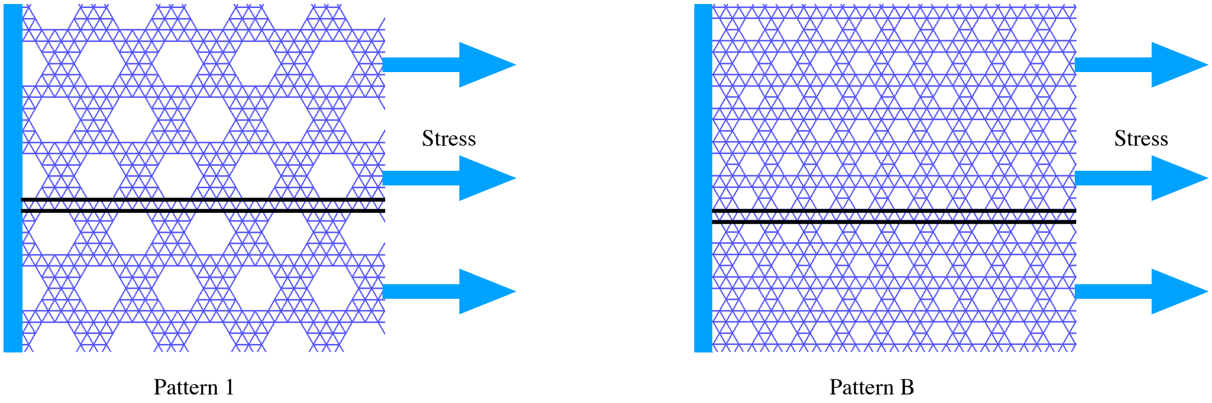

Supplementary Figure 2. Different responses to uniaxial stresses. Diagram illustrating the different response of two patterns with similar amount of removed area ($s = 0.44$ for Pattern 1 and $s = 0.47$ for Pattern B). For both cases we apply a uniaxial (horizontal) stress. Now, as one can see, in these $30a \times 30a$ sections of the complete systems, Pattern 1 has 6 horizontal linkages, while Pattern B has 11 (we highlight one in each case). Therefore, we expect their Young's moduli to be $11Y_{\text{eff}}^{(1)} \approx 6Y_{\text{eff}}^{(B)}$.

The observed functional dependence of T_c on the fraction of removed area $kT_c/\tilde{\kappa} = A(1-s)^c$ cannot be explained just by evaluating the $T = 0$ elastic constants for perforated membranes. To see this, let us show that the $T = 0$ bending rigidity is linear in the fraction of removed area. We first note that the total energy at $T = 0$ is a sum of positive-definite terms. It is therefore always minimized by setting all the springs to their rest length and the bending energy to zero. The energy of a discretized full sheet is $E_{\text{bending}} = E = \tilde{\kappa} N_{\text{dih}} E_0$, where E_0 is the energy of each dihedral and N_{dih} is the number of dihedrals. In order to write the energy in terms of the area, we note that the total area is just the sum of the areas of the individual triangles, $S = N_{\text{tri}} S_{\text{tri}}$. Now $N_{\text{dih}} = 3N_{\text{tri}}/2$, so we can write $E = \frac{3}{2}\tilde{\kappa} N_{\text{tri}} E_0 = \frac{3}{2}\tilde{\kappa} E_0 S/S_{\text{tri}}$. Taking the continuum limit we find $\kappa \propto E/S$. When we remove area the energy will decrease linearly in S, which can be interpreted as a κ_{eff} decreasing linearly with S while keeping the original intact area fixed. If κ_{eff} were to determine the crumpling temperature it would be linear in $(1-s)$ rather than decreasing as $(1-s)^{1.93}$.

We could instead consider the response of perforated membranes to uniaxial stresses, which at $T = 0$ will be characterized by a Y_{eff}. In this case, we do expect the elastic response to be very sensitive to the details of the hole arrangement, leading to a non-linear dependence of Y_{eff} on the geometry. We do not, however, expect Y_{eff} to be relevant for the crumpling transition. Indeed, we

know from the theory of thermalized elastic membranes that the Young's modulus only affects T_c as a logarithmic correction [see Eq. (9) in the main text].

This can be illustrated with an example from our simulations by looking closely at Patterns 1 and B. Looking at Fig. 3., one will notice that these two geometries have a very similar κ/kT_c. Even though they look very different, counting the number of remaining triangles we see that the total fractions of removed area are very close ($s = 0.44$ for Pattern 1 and $s = 0.47$ for Pattern B). If we consider, on the other hand, a stress applied in the horizontal direction at $T = 0$, as in Supplementary Fig. 2, we see that their response will be significantly different. Indeed, counting the number of horizontal linkages in each pattern we can estimate $11Y_{\text{eff}}^{(1)} \approx 6Y_{\text{eff}}^{(B)}$, a difference that does not reflect in their respective T_c.

Notice, finally, that for anisotropic geometries such as Pattern 3 (Fig. 9), the crumpling transition is at the same temperature we would expect for an isotropic pattern with the same removed area, further reinforcing the result that the elastic response to uniaxial stress is essentially irrelevant.

SUPPLEMENTARY NOTE 2: THE THERMAL CRITICAL EXPONENT

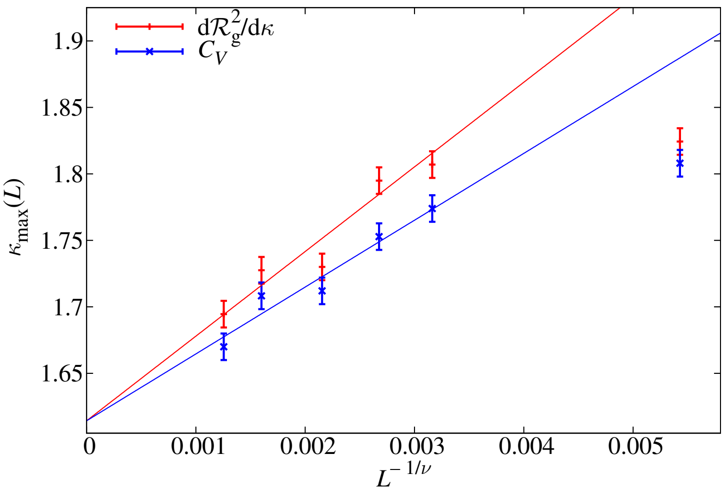

Supplementary Figure 3. The thermal critical exponent. We show the scaling of the position of the peaks for the specific heat and the $\tilde{\kappa}$-derivative of the gyration radius. We plot the position of each peak against $L^{-1/\nu}$, with $\nu = 0.73$ from [1]. We then perform linear fits for $L \geq 75$, forcing both curves to extrapolate to the same $\tilde{\kappa}/kT_c$.

As discussed in the main text, a reliable direct computation of the thermal critical exponent ν is not possible with our data. In principle, one could study the evolution of the position of the maximum for either C_V or $\mathrm{d}\mathcal{R}_g^2/\mathrm{d}\tilde{\kappa}$. According to standard finite-size scaling [2], this quantity should shift with system size as

$$\left(\frac{\tilde{\kappa}}{kT}\right)_{\max} = \tilde{\kappa}/kT_c + AL^{-1/\nu} + \dots\,, \tag{1}$$

where the dots represent corrections to leading scaling for large L. Unfortunately, for this quantity these corrections are very strong (even for unperforated lattices [3]), so in a fit to (1) we would have to discard several system sizes. This, together with the large fluctuations in the peaks' positions and the fact that we would have to fit both for the asymptotic value and for the exponent, prevents us from evaluating ν directly. We can, however, verify that the known value for the standard crumpling transition ($\nu = 0.73$ [1, 3]) is compatible with our data. This is shown in Supplementary Fig. 3.

[1] Le Doussal, P. & Radzihovsky, L. Self-consistent theory of polymerized membranes. *Phys. Rev. Lett.* **69**, 1209–1212 (1992).

[2] Amit, D. J. & Martin-Mayor, V. *Field Theory, the Renormalization Group, and Critical Phenomena* (World Scientific, Singapore, 2005), 3rd edn.

[3] Cuerno, R., Gallardo Caballero, R., Gordillo-Guerrero, A., Monroy, P. & Ruiz-Lorenzo, J. J. Universal behavior of crystalline membranes: Crumpling transition and poisson ratio of the flat phase. *Phys. Rev. E* **93**, 022111 (2016).
