# Supplementary material for: Thermal crumpling of perforated two-dimensional sheets
Source: Nat Commun. 2017 Nov 9;8:1381. doi: 10.1038/s41467-017-01551-y (PMC5680302; doi:10.1038/s41467-017-01551-y)
Supplement: Supplementary file 3 — Description of Additional Supplementary Files [file 41467_2017_1551_MOESM3_ESM.pdf]

Description of Additional Supplementary Files

File Name: Supplementary Movie 1
Description: Comparison of pristine and perfected membranes for $kT = 1.14\,\tilde{\kappa}$
Superimposed animations of a full (magenta) and a perforated (cyan) membrane for a temperature (in units of the bending rigidity) of $kT = 1.14\,\tilde{\kappa}$. The full membrane is in the extended phase, while the perforated membrane is starting to crumple.

File Name: Supplementary Movie 2
Description: Comparison of pristine and perforated membranes for $kT = 1.25\,\tilde{\kappa}$
Superimposed animations of a full (magenta) and a perforated (cyan) membrane for a temperature (in units of the bending rigidity) of $kT = 1.25\,\tilde{\kappa}$. The full membrane is in the extended phase, while the perforated membrane has crumpled.
